# Patterns of chromosome evolution in ruminants

**DOI:** 10.1111/mec.17197

**Published:** 2023-11-08

**Authors:** Cristina Arias‐Sardá, Sarah Quigley, Marta Farré

**Affiliations:** ^1^ School of Biosciences University of Kent Canterbury UK

**Keywords:** Comparative genomics, Genomics/Proteomics, Mammals, Molecular Evolution, Chromosome evolution

## Abstract

Studying when and where gross genomic rearrangements occurred during evolution is key to understanding changes in genome structure with functional consequences that might eventually lead to speciation. Here we identified chromosome rearrangements in ruminants, a clade characterized by large chromosome differences. Using 26 genome assemblies, we reconstructed five ancestral karyotypes and classified the rearrangement events occurring in each lineage. With these reconstructions, we then identified evolutionary breakpoints regions (EBRs) and synteny fragments. Ruminant karyotype evolution is characterized by inversions, while interchromosomal rearrangements occurred preferentially in the oldest ancestor of ruminants. We found that EBRs are depleted of protein coding genes, including housekeeping genes. Similarly, EBRs are not enriched in high GC regions, suggesting that meiotic double strand breaks might not be their origin. Overall, our results characterize at fine detail the location of chromosome rearrangements in ruminant evolution and provide new insights into the formation of EBRs.

## 1 INTRODUCTION

**FIGURE 1:**
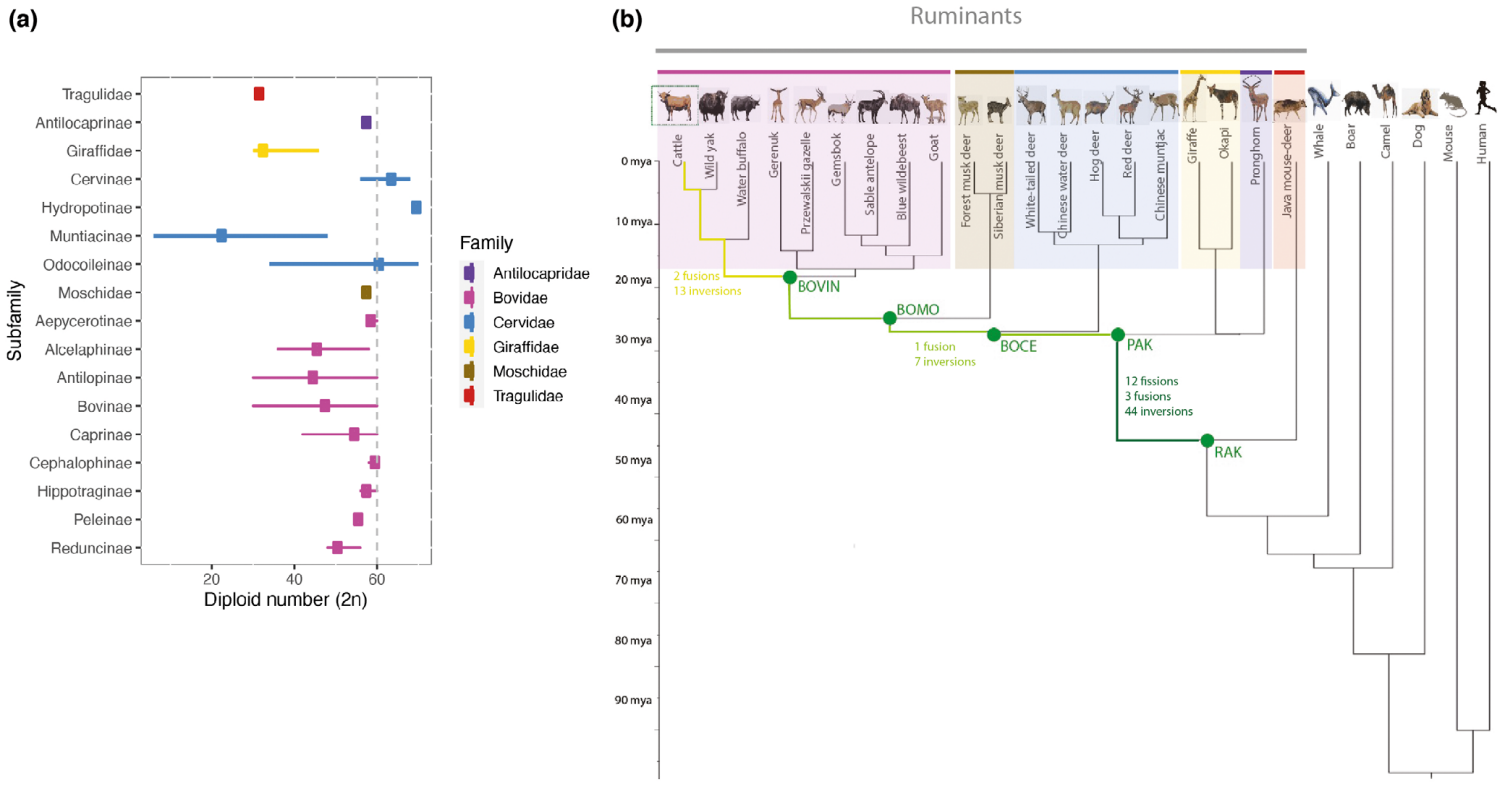
Ruminant species. (a) Chromosome numbers (2*n*) of ruminant families and subfamilies. (b) Phylogenetic tree of the species included in the analysis. The different shades of green in the branches indicate the ancestral karyotypes reconstructed and the number and type of chromosome rearrangements between each ancestor. BOCE, ancestral karyotype of the Cervidae, Moschidae and Bovidae; BOMO, ancestral karyotype of Moschidae and Bovidae; BOVIN: bovid ancestral karyotype; PAK, pecoran ancestral karyotype; RAK, ruminant ancestral karyotype.

Ruminantia, the largest suborder within the Cetartiodactyla order of mammals, includes 234 species (Burgin et al., 2018; Mammal Diversity Database, 2022) in two infraorders, Tragulina and Pecora, that diverged ~50 million years ago (Fernández & Vrba, 2005; Zachos et al., 2001). Ruminant species belong to six families (Tragulidae, Antilocapridae, Giraffidae, Cervidae, Moschidae and Bovidae), with around 23% of species being endangered or critically endangered in the IUCN Red List (IUCN, 2022) (Table S1). Ruminants exhibit extreme morphological and ecological characteristics such as a multichambered stomach, cranial appendages (headgear) (Davis et al., 2011), specialized dentition, a highly cursorial locomotion and a wide range of body size variations. Ruminants are distributed across extensive habitats, including different altitudes, latitudes and ecological environments (Wang et al., 2019). Alongside, ruminants show a wide variation in diploid chromosome numbers, from 2*n* = 6/7 to 2*n* = 70 in the Indian muntjac (*Muntiacus muntjak*) and white‐tailed deer (*Odocoileus virginianus*), respectively, with most of the species having 2*n* = 60 chromosomes, especially in the Bovidae family (Figure 1a) (Graphodatsky et al., 2020). This disparity in chromosome number is due to chromosome rearrangements and not changes in ploidy.

Chromosome rearrangements (CRs) can facilitate adaptation (Joron et al., 2011; Mérot et al., 2021), change gene expression patterns of nearby genes (Berdan et al., 2021; Farré et al., 2019; Lazar et al., 2018) and alter recombination (Farré et al., 2013; Navarro & Barton, 2003) which could eventually lead to speciation (reviewed in (Damas et al., 2021)). Comparative genomic studies in mammals and birds have shown that syntenic fragments (SFs, regions of the genomes with the same order of markers) and evolutionary breakpoint regions (EBRs, localized at the end of SFs) occurred in distinct genomic contexts (Farré et al., 2016; Larkin et al., 2009; Murphy et al., 2005). SFs are enriched for evolutionary conserved sequences and genes related to basic organismal development (Farré et al., 2016; Larkin et al., 2009); instead, EBRs are clustered in regions with transposable elements (Farré et al., 2011, 2019; Larkin et al., 2009) and collocate with genes related to lineage‐specific biology (Farré et al., 2019; Kim et al., 2017). However, the evolutionary forces that lead to the appearance and fixation of CRs are still unclear.

CRs have been widely studied in ruminants, mostly using cytogenetic and genetic maps (Kulemzina et al., 2009, 2011; Slate et al., 2002). However, the resolution of these approaches is limited, hindering the identification of EBRs and SFs. Recently, with the implementation of DESCHRAMBLER (Kim et al., 2017), we reconstructed the ancestral karyotype of three ruminant ancestors using nine ruminant genome assemblies. We showed that ruminant karyotypes are populated with multiple inter‐ and intrachromosomal rearrangements that might affect gene expression and regulation in the lineage leading to cattle (Farré et al., 2019). Here, we expand on our previous work by generating five new in silico karyotype reconstructions using 20 ruminant genomes including at least one representative of each family (Figure 1b). This allows us to refine the identification and timing of CRs in ruminant evolution. Looking at EBRs and SFs we also point to possible mechanisms in the formation and fixation of CRs. Our results significantly increase knowledge of ruminant genome evolution and will facilitate greater understanding of the role of chromosome rearrangements in adaptation and speciation in this lineage, as well as the possible mechanisms leading to their formation.

## 2 MATERIALS AND METHODS

### 2.1 Genome selection and filtering

Details of all publicly available genome assemblies, assembly quality and known diploid number in all extant ruminant species can be found in Table S1 (Dudchenko et al., 2017; Foley et al., 2023; Graphodatsky et al., 2020). A total of 20 species with genomes assembled at chromosome‐level or with a scaffold N50 ≥ 1 Mbp, and representing all ruminant families and subfamilies, were included for further analysis (Table 1). Moreover, six outgroup species (human, mouse, dog, pig, camel and sperm whale) were also included in the analysis. All genome assemblies were filtered using faFilter from UCSC Kent Utilities (Kent et al., 2010; Kuhn et al., 2013) to remove all scaffolds smaller than 10,000 bp ensuring unplaced scaffolds were not included in the analysis. Genome assemblies obtained from NCBI (Table 1) were already soft‐masked and no further filters were done, while genomes downloaded from DNA zoo were soft‐masked using RepeatMasker version 4.0.9 (Tempel, 2012) with the same options for all species (−pa −64 ‐xsmall ‐species cattle).

**TABLE 1:** Genome assemblies used in this study.

Common name	Scientific name	Assembly ID/GeneBank accession number	Assembly level	Scaffold N50 (Mbp)	Size (Gb)	^a^% SFs
Pronghorn	*Antilocapra americana*	AntAmePen_v2_BIUU_UCD/GCA_004027515.2	Scaffold	18.8	2.96	94.63
Wild yak	*Bos mutus*	ASM764659v3/GCA_007646595.3	Scaffold	16.6	2.76	94.44
Water buffalo	*Bubalus bubalis*	UOA_WB_1/GCA_003121395.1	Scaffold	117.2	2.66	91.84
Goat	*Capra hircus*	ARS1/GCA_001704415.2	Chromosome	N/A	2.92	93.88
Blue wildebeest	*Connochaetes taurinus*	BWD_HiC/N/A	Chromosome	N/A	2.65	95.91
Sable antelope	*Hippotragus niger*	Sable_antelope_Masurca.scf_HiC/N/A	Chromosome	N/A	2.6	95.99
Gerenuk	*Litocranius walleri*	GRK_HiC/N/A	Chromosome	N/A	2.98	94.99
Gemsbok	*Oryx gazella*	UCDavis_Ogaz_1/GCA_003945745.1	Chromosome	N/A	2.74	84.57
Przewalski's gazelle	*Procapra przewalskii*	PLG/GCA_006410515.1	Scaffold	5.5	2.69	89.3
Hog deer	*Axis porcinus*	ASM379854v1_HiC/N/A	Chromosome	N/A	2.68	95.87
Red deer	*Cervus elaphus*	CerEla1.0/GCA_002197005.1	Chromosome	N/A	3.44	95.24
Chinese water deer	*Hydropotes inermis*	NPU_HINE_1.0/GCA_006459105.1	Scaffold	13.8	2.53	94.25
Chinese muntjac	*Muntiacus reevesi*	CIJ_HiC/N/A	Chromosome	N/A	2.6	95.74
White‐tailed deer	*Odocoileus virginianus*	Ovir.te_1.0_HiC/N/A	Chromosome	N/A	2.38	95.67
Giraffe	*Giraffa camelopardalis*	Giraffe.scafSeq.fill.gapcloser/N/A	Chromosome	N/A	2.44	95.81
Okapi	*Okapia johnstoni*	ASM166083v1_HiC/N/A	Chromosome	N/A	2.89	95.59
Forest musk deer	*Moschus berezovskii*	ls35.final.genome_HiC/N/A	Chromosome	N/A	2.73	95.84
Siberian musk deer	*Moschus moschiferus*	MosMos_v2_BIUU_UCD/GCA_004024705.2	Scaffold	11.7	2.11	90.87
Java mouse‐deer	*Tragulus javanicus*	ASM402496v2/GCA_004024965.2	Scaffold	14.1	2.59	92.95

^a^ Coverage syntenic fragments (SFs) of the pair‐wise alignments of each species to cattle genome.

### 2.2 Establishing ancestral karyotypes

To define ancestral karyotypes, we used DESCHRAMBLER, a tool to reconstruct ancestral karyotypes (Kim et al., 2017). This tool takes as input a phylogenetic tree and pairwise alignments of the species included in the analysis.

#### 2.2.1 Pairwise alignments

Pairwise alignments were performed using the cattle genome (*Bos taurus*) as a reference. We used lastZ version 1.04.00 (Harris, 2007) with the following parameters: ‐minScore = 1000, *C* = 0, *E* = 30, *K* = 3000, *L* = 3000, *O* = 400. The output of lastZ was then converted into chain and net files using the following UCSC Kent Utilities: axtChain (parameters: ‐psl ‐verbose = 0 ‐minScore = 1000 ‐linearGap = medium), chainAntiRepeat, chainSort, chainPreNet chainNet and netSyntenic. Syntenic fragments (SFs) of 150‐Kbp and 300‐Kbp resolution were represented in Evolution Highway (EH) images using syntenyPlotteR (Farré et al., 2019). The coverage of the net files was calculated to assess reliability of the alignment (Table 1).

#### 2.2.2 Phylogenetic tree

A phylogenetic tree including all 20 ruminant and six outgroup species was obtained using TimeTree divergence time estimates (Kumar et al., 2017), written in newick format and plotted using FigTree (Rambaut, 2009).

#### 2.2.3 DESCHRAMBLER analysis

We started the reconstruction running DESCHRAMBLER (Kim et al., 2017) with a resolution of 300 Kbp and minimum adjacency score of 0.01. Five different ancestors were reconstructed: ruminant ancestor (RAK), pecoran ancestor (PAK), Cervidae+Moschidae+Bovidae ancestor (BOCE), Moschidae+Bovidae ancestor (BOMO) and Bovidae ancestor (BOVIN). The reconstructed ancestral chromosome fragments produced by DESCHRAMBLER were manually curated and merged to reconstruct ancestral chromosomes using previous studies as reference (Farré et al., 2019). For RAK and PAK reconstruction, the number of ancestral chromosome fragments reconstructed by DESCHRAMBLER was higher than the number of chromosomes previously proposed by these studies. Previous studies have shown that occasionally ancestral chromosomes are more fragmented than what has been suggested by other methodologies, in part because the use of scaffold assemblies of descendant species, where the exact tips of chromosomes are not known, and/or in part because of ambiguous cases resulting from insufficient evidence of adjacency (Kim et al., 2017). We manually curated our reconstructions by merging ancestral fragments following the structure of FISH‐based reconstructions (Farré et al., 2019), and orientating of these merged ancestral fragments into ancestral chromosomes by minimizing the number of rearrangements. In some instances, ancestral chromosome fragments were not merged into larger ancestral chromosomes, due to the inability to manually resolve complex rearrangements. In these cases, ancestral chromosomes were formed by more than one fragment, and labelled accordingly to their size. Visualizations of ancestral karyotypes were made using syntenyPlotteR (Farré et al., 2019).

### 2.3 Detection of chromosome rearrangements (CRs) and evolutionary breakpoint regions (EBRs)

We classified CRs between ancestors into fusions, fissions, inversions and complex rearrangements (when more than one type of CR occurs). We then defined evolutionary breakpoint regions (EBRs) as the breaks of synteny at the edges of CRs in each of the ancestors. EBRs were then classified by size into well‐defined EBRs (ranging from 0 bp to 50 Kbp) and not defined EBRs (ranging from 50 Kbp to 300 Kbp). When the break of synteny was >300 Kbp, these regions were considered gaps and discarded from our analysis. The EBRs were then phylogenetically classified depending on the ancestral lineage in which they occurred into ancestor specific or reference species specific. Finally, EBRs were further separated by the type of rearrangement they delimited into inversion EBRs or interchromosomal EBRs, if they demarcated inversions or were the result of fusion or fission events respectively.

Syntenic fragments (SFs) forming CRs were classified as collinear, inversion, fusion or fission SFs depending on whether they have been conserved syntenic during evolution (collinear SFs) or are the result of any type of chromosome rearrangement that change their position during evolution (Figure S1).

### 2.4 Identification of housekeeping genes

Normalized gene expression data in transcripts per million (TPM) were downloaded on 23/05/23 from the Gene Expression Atlas (https://www.ebi.ac.uk/gxa/home (Moreno et al., 2022)) for cow (Merkin et al., 2012), pig (Eory, 2017) and sheep (Jiang et al., 2014), whereas gene expression data for human were downloaded from https://gtexportal.org/home/datasets GTEx release V8 (dbGap Accession phs000). Only tissues present in all four species were used for further analysis. We considered as housekeeping genes those genes expressed at a constant or stable level in almost all tissues of the dataset, following previous publications (Eisenberg & Levanon, 2013). First, we labelled genes as expressed if they have >10 TPM in over 90% of the tissues in each species (Papatheodorou et al., 2019). Then, we calculated whether genes were stably expressed across tissues using the nonparametric metric Gini coefficient, traditionally used in economics for determining income inequality, and previously used to assess expression inequality for transcriptomic data (Jiang et al., 2018). The Gini coefficient provides a value between 0 and 1 for each gene, representative of the expression equality of a given gene. The closer the value to 1 the greater the expression inequality becomes. We used the function Gini from DEscTools v 0.99.31 (Signorell, 2023). Only genes with a Gini coefficient < = 0.4 were considered housekeeping genes.

To cross‐compare housekeeping genes across species, we downloaded single copy orthologous genes for the four species from Ensembl (Cunningham et al., 2022). We considered mammalian ancestral housekeeping genes those orthologs that were labelled as housekeeping in all species, while ruminant ancestral housekeeping genes only those orthologs that were labelled as housekeeping in cattle and sheep only. Finally, any housekeeping genes in the cattle genome not identified as either mammalian or ruminant ancestral or housekeeping in any other species were named cattle‐specific housekeeping genes. An UpsetR plot to visualize this relationship was created using UpSetR (version 1.4.0) in R (version 4.2.2).

### 2.5 Statistical analysis

To assess the relationship between EBRs and SFs with genomic and transcriptomic features, we downloaded the unique protein coding genes and transposable elements from the cattle genome ARS‐UCD1.2 using Ensembl BioMart. We calculated the fraction of GC content in windows of 10 Kbp in the cattle genome using bedtools nuc (Quinlan & Hall, 2010). Windows with at least 60% GCs were labelled as high GC content. This cut off was chosen because it represents two times the 95% confidence interval of the mean % GC in 10Kbp windows of the genome. We then used *RegioneR* R package version 1.26 with 1000 permutations in each dataset to evaluate the overlap of these features, with a *p*‐value ≤.05 considered statistically significant.

We assessed the density of these features in regions surrounding the well‐defined EBRs. First, we calculated the density of the genomic features (as number of bases of the feature) in non‐overlapping 10 Kbp windows in the cattle genome using bedtools coverage (Quinlan & Hall, 2010). Then 30 10Kbp windows up‐ and down‐stream of each EBR midpoint were selected, and the mean density of each window was calculated using a custom script. We finally plotted the density in a composite plot using ggplot2 (version 3.3.5) in R (version 4.1.0).

An additional analysis was performed to study the correlation of the EBRs and the TEs content of cattle. Genomic Association Test (GAT) version 1.3.4 (Heger et al., 2013) was used to compute the significance of overlap between EBRs and different families of TEs. A *p*‐value ≤.05 was considered statistically significant.

## 3 RESULTS

To assess the functional contribution of chromosomal rearrangements to the evolution of ruminants, we first reconstructed ruminant ancestral karyotypes using 20 ruminant genomes including at least one representative of each ruminant family (Table 1, Figure 1). Five ruminant ancestral karyotypes were reconstructed: RAK, the deepest node, ancestral to all ruminants; PAK, the pecoran ancestral karyotype; BOCE, the ancestral karyotype that included Cervidae, Moschidae and Bovidae families; BOMO, the common ancestral karyotype including Moschidae and Bovidae families; and BOVIN, the shallowest node, ancestral of the Bovidae family.

### 3.1 Reconstructing ancestral karyotypes

We used DESCHRAMBLER to reconstruct the five ancestors with a resolution of 300 Kbp and an adjacency score of 0.01. All ancestral reconstructions showed high genome coverage of the reference genome (cattle), ranging from 88.6% to 93.93% in the deepest node RAK and the shallowest node BOVIN respectively. DESCHRAMBLER generated a total of 32 and 40 ancestral chromosome fragments for PAK and RAK respectively, while 30 ancestral chromosome fragments for BOCE, BOMO and BOVIN were generated. We then manually curated the ancestral chromosome fragments to increase coverage and minimize overestimation of CRs (see Methods). After the curation, a total of 27 ancestral chromosome fragments were obtained in RAK and 30 in the rest of ancestors (Table 2).

**TABLE 2:** DESCHRAMBLER results. No. of chromosomes (*n*) indicates the haploid number of our reconstructions.

Ancestor	No. RACFs	No. chrom (*n*)	Max. Size (kb)	Min. Size (kb)	Total size (Gb)	Ref. coverage^a^
RAK	27	23 + X	192,591	1398	2.41	88.6
PAK	30	28 + X	151,319	855	2.45	90.35
BOCE	30	28 + X	151,973	1579	2.47	91.05
BOMO	30	28 + X	154,317	7360	2.49	91.18
BOVIN	30	28 + X	184,490	365	2.53	93.83

^a^ Coverage against cattle genome (size = 2.71Gb). RACFs: reconstructed ancestral chromosome fragments.

**FIGURE 2:**
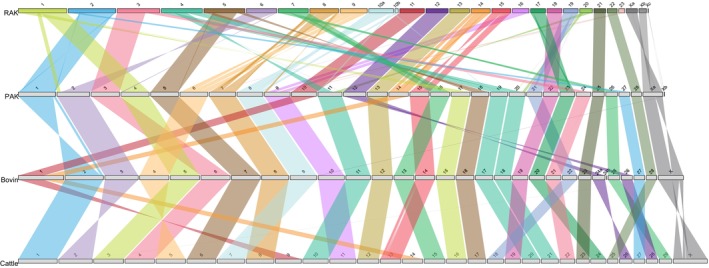
Comparison of reconstructed ancestral karyotypes. Colours indicate different chromosomes, with ribbons showing syntenic relationships. Inversions are shown when ribbons are twisted. BOVIN, bovid ancestral karyotype; PAK, pecoran ancestral karyotype; RAK, ruminant ancestral karyotype.

Most of the ancestral chromosome fragments represent entire ancestral chromosomes, except in six ancestral chromosomes (Figure 2). RAK, the deepest node, presents a haploid number of 23 + X within 27 fragments, with RAK10 and RAKX comprised of two and three fragments each respectively. PAK consists of 30 ancestral chromosome fragments, with PAKX split into two fragments, showing the same haploid number (28 + X) that the Pecora ancestor reported previously (Farré et al., 2019). BOCE and BOMO contain 30 ancestral chromosome fragments with a haploid number of 28 + X in both ancestors, due to the fragmentation of BOCEX and BOMO11. Finally, BOVIN, the shallowest node, presents 30 ancestral chromosome fragments, with BOVIN24 fragmented into two pieces. Our BOVIN reconstruction presents a lower haploid number (28 + X) than previously reported (29 + X), due to BOVIN1 including the fusion of cattle chromosomes 9 and 14 (Farré et al., 2019).

### 3.2 Detection of chromosome rearrangements (CRs) and evolutionary breakpoint regions (EBRs) in ruminants

Ancestral karyotypes provide the basis to identify and date CRs that occurred during ruminant evolution. To this end, we cross‐compared SFs in each ancestral reconstruction and used parsimony to delineate the CRs between RAK and PAK, PAK and BOVIN; and BOVIN and cattle (Figure 2 and Table S2). Only six chromosomes were maintained collinear between RAK and PAK (RAK 11, 12, 13, 18, 21 and 22), while a total of 59 CRs occurred, with 12 fissions, three fusions, and 44 inversions (Figure 2), representing a CR rate of 0.94 interchromosomal rearrangements/million years (My), 2.75 inversions/My, and a total of 3.69 CRs/My. Contrariwise, only eight CRs differentiated PAK from BOVIN, with one fusion and seven inversions (Figure 2), indicating that intrachromosomal rearrangements were the main structural change between PAK to BOVIN genome. Additionally, twenty‐one ancestral chromosomes remained collinear between PAK and BOVIN (PAK chromosomes 2–5, 7, 8, 11, 13, 15–28). This translates to 0.1 and 0.7 interchromosomal rearrangements and inversions/My, respectively, and a total of 0.8 CRs/My between PAK and BOVIN. Finally, the youngest ancestral karyotype reconstructed, BOVIN (Figure 2), differs from cattle in two fusions and 13 inversions, with 0.11, 0.72 and 0.83 of interchromosomal, inversion and total CRs/My of evolution respectively. Overall, only four RAK chromosomes, homologous to cattle chromosomes BTA12, 19, 23 and 25, were maintained completely syntenic for 50 million years of evolution (Figures S2 and S3).

We then classified SFs in the cattle genome depending on the type of rearrangement that occurred since the divergence of RAK into: (i) collinear, when SFs have been conserved between species, (ii) inversion, (iii) fission, (iv) fusion or (v) a combination of some of the previous cases (Figure S1). A total of 48.84% of cattle genome has been maintained collinear, 8.77% inverted, and 40.48% into interchromosomal rearrangements (specifically, 17.74% fissioned and collinear SFs, 21.37% fissioned and fused SFs, and 1.67% fissioned and inverted SFs). These results highlight the important role that interchromosomal rearrangements have played in genome reshuffling during Ruminantia species evolution.

CRs are by definition delimited by evolutionary breakpoint regions (EBRs). Looking at all CRs placed in the reference genome context, we found a total of 56 EBRs in cattle (Table S3). To study their location and genomic characteristics, we first divided them into well‐defined if their genomic size is 0–50 Kbp or not defined if their size is 50–300 Kbp. A total of 32 and 24 were well defined and not defined EBRs respectively. The EBRs were then classified depending on whether they were cattle or ancestor specific. Within the 32 well defined EBRs, three were cattle‐specific, three BOVIN‐specific, two PAK‐specific and 24 RAK‐specific. Within the 24 not defined EBRs, five were cattle‐specific, one PAK‐specific and 18 RAK‐specific. EBRs, including all types, are not uniformly distributed in cattle chromosomes (Figure S4). Cattle chromosome 11 and 21 showed a significantly higher density of EBRs (0.056, 0.057 EBRs/Mbp respectively) than the genomic mean of 0.021 EBRs/Mbp (*Z*‐score 2.09, *p*‐value = .03; and *Z*‐score 2.16, *p*‐value = .03, respectively, Figure S4). Contrariwise, no type of EBRs were located in cattle chromosomes 4, 6, 9, 12, 19, 23, 25 and 27.

### 3.3 Assessing the non‐random distribution of CRs and EBRs

**FIGURE 3:**
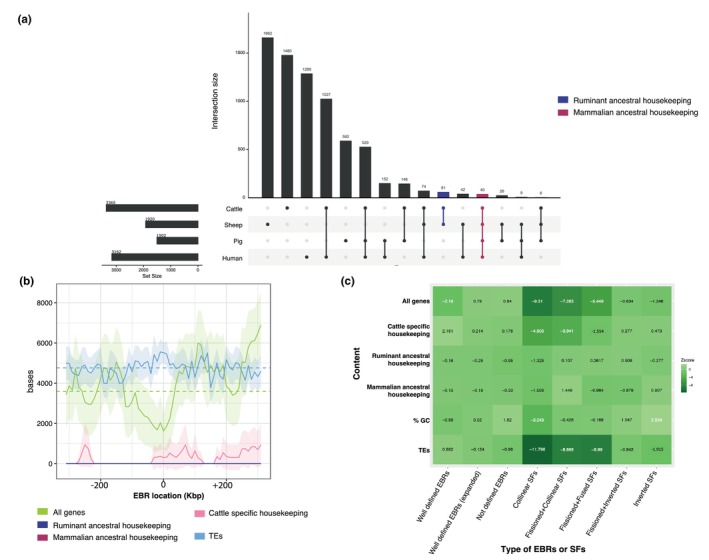
Association of evolutionary breakpoint regions (EBRs) with several genomic and transcriptomic data. (a) Overlap of housekeeping genes in four mammalian species. The number of housekeeping genes in each species is shown in the left barplot as ‘set size’. The x‐axis represents the number of genes being housekeeping for the different overlap combinations of species. Different combinations of overlap are represented by lines interlinking dots, while the bar chart on top shows the degree of overlap as ‘intersection size’. In red, we show the mammalian ancestral housekeeping genes, while in blue the ruminant ancestral housekeeping genes. (b) Composite plot showing the density of genomic features in regions surrounding well defined EBRs. Dark coloured lines indicate the mean density (number of bases of each feature in 10Kbp windows) up‐ and down‐stream of the midpoint of EBRs, while coloured shading shows the 95% confidence intervals. Dotted lines indicate the genome‐wide mean of each feature. (c) Heatmap plotting the *z*‐score of the statistical association of EBRs and SFs with genomic and transcriptomic data. Shading indicates the *z*‐score value, while white numbers show significant associations (*p*‐value ≤.05).

Seeing the pattern that EBRs and CRs are not uniformly distributed across ruminant chromosomes, we studied whether they occur in non‐random locations of the genome. We previously hypothesized that DNA sequence composition, the 3D structure of the chromatin within the nucleus and the effect on gene expression were key elements in determining the genomic distribution of EBRs, as part of the Integrative Breakage Model (Farré et al., 2015, 2019). Here, we tested this model by first looking at the genomic and genic content within and nearby EBRs. To do so, several permutation tests were performed over three different sets of EBRs: (i) well‐defined EBRs; (ii) not defined EBRs; and (iii) regions near well‐defined EBRs (±30 Kbp) (Figure 3). Only 8 out of 21,861 protein coding genes annotated in cattle are found within well‐defined EBRs, representing a clear negative association between genes and well‐defined EBRs (*Z*‐score = −2.191 and *p*‐value = .0195, Figure 3b,c). In not defined EBRs, 294 protein‐coding genes were found, while 48 genes were in surrounding regions of well‐defined EBRs; however, these overlaps are not statistically significant (*Z*‐score = .84 and *p*‐value = .278; *Z*‐score = .79 and *p*‐value = .259, respectively, Figure 3b,c). Therefore, these results indicate that well defined EBRs do not co‐localize with genes (Figure 3b).

Previous publications suggested that CRs might be the result of non‐allelic homologous recombination between segmental duplications or transposable elements. Here, we used permutation testing to assess the overlap of EBRs and transposable elements. Although well‐defined EBRs are not enriched in transposable elements overall (*Z*‐score = .882, *p*‐value = .259), several ruminant‐specific transposable elements are significantly associated with EBRs, including ERV1, ERV2 and BovB (Figure S5).

Because CRs must start with an incorrectly repaired double strand break (DSB) and to be passed to the next generation this must happen in the germline, it has been suggested that EBRs might co‐localize with crossovers in meiosis. Here, we used high GC content as a measure of past recombination levels linked to biased gene conversion to assess if EBRs are related to meiotic crossovers. A total of 2353 windows contained more than 60% of GC, representing 8.66% of the cattle genome. Contrary to our expectation, EBRs are not associated with high GC windows in the cattle lineage (well‐defined EBRs *Z*‐score = −.691, *p*‐value = .6214, not defined EBRs *Z*‐score = 1.62, *p*‐value = .124, Figure 3c); whereas collinear SFs are negatively associated with high GC content (*Z*‐score = −3.249, *p*‐value = .003) and inverted SFs are enriched (*Z*‐score = 2.554, *p*‐value = .023).

The Integrative Breakage Model predicts that only those reorganizations not disturbing essential genes and/or gene expression would likely be the only ones fixed within populations. To test this expectation, we defined housekeeping genes in four species, including cattle, sheep, pig and human (Figure 3a). This was performed using publicly available gene expression data, filtered to include only tissues from organs found in the datasets for all species (brain, colon, heart, kidney, liver, lung, skeletal muscle, spleen and testis) improving comparability of the results. Available samples were taken from various structures within the organs thus leading to variable tissue quantities across the species: nine, 13, 17, and 24 samples from cow, sheep, pig and human respectively. We then obtained single copy orthologous genes for all four species from Ensembl (Cunningham et al., 2022). We defined mammalian ancestral housekeeping genes, as those orthologs being housekeeping in all four species; or ruminant ancestral housekeeping, as those orthologs considered housekeeping in only cattle and sheep (Figure 3a). A total of 40 and 61 housekeeping genes were labelled as mammalian or ruminant ancestral, respectively, while 1480 were cattle‐specific (Figure 3a and Table S4).

Overall, ~2% of all housekeeping genes co‐located with EBRs while ~98% were within SFs (Table S5). Mammalian and ruminant ancestral housekeeping genes are not found in any type of EBRs, and only one and 30 cattle‐specific housekeeping genes co‐locate with well‐defined and not defined EBRs, respectively, although not statistically significant (Figure 3c). Mammalian and ruminant ancestral housekeeping genes are mainly located in collinear SFs (52.5% and 37.7% respectively) and fissioned‐fused SFs (27.5% and 36.06%, respectively, Tables S4 and S5). But because collinear and fissioned‐fused SFs represent 48.84% and 21.37% of the cattle genome, these overlaps are not statistically significant (collinear SFs *Z*‐score = −1.32, *p*‐value = .16, and *Z*‐score = −1.5, *p*‐value = .13 for ruminant and mammalian ancestral genes, respectively, Figure 3c). Instead, 50.81% of cattle‐specific housekeeping genes are located in collinear SFs; representing a significant depletion in these genomic regions (*Z*‐score = −4.81, *p*‐value = .001). Similarly, a negative association between cattle‐specific housekeeping genes and fission+collinear SFs was found (*Z*‐score = −3.94, *p*‐value = .001, Figure 3c).

## 4 DISCUSSION

Understanding the mechanisms and impact of genomic rearrangements has been an active area of research since the first comparative cytogenetic maps were described. Now, with the availability of chromosome‐scale genome assemblies generated by large consortiums (Foley et al., 2023; Rhie et al., 2021), this area of research is again at the forefront of evolutionary biology. Here, we assessed the patterns of chromosome evolution in ruminants, a clade characterized by extreme differences in diploid numbers (Figure 1a). We first reconstructed five ancestral karyotypes using 26 genome assemblies: the ancestor of all ruminant families (RAK), the ancestor of Pecoran species (PAK), the ancestor of Cervidae, Moschidae and Bovidae families (BOCE), the ancestor of Moschidae and Bovidae families (BOMO), and the ancestor of Bovidae family, the shallowest node (BOVIN). After manual curation, RAK, the deepest node, comprised 23 + X chromosomes with RAK10 and RAKX split into two and three fragments respectively (Figure 2). This fragmentation, also present in PAKX and BOVIN24, is most likely due to a lack of support from the outgroup species, making DESCHRAMBLER unable to join these fragments. Poor alignment with the outgroup species and/or misassemblies in ingroup genomes in these areas might also cause this issue. Our reconstructions mostly agree with in silico ancestral karyotypes previously published (Damas et al., 2022; Farré et al., 2019), except in the fusion of cattle chromosomes 9 and 14 that we reported in BOVIN. This is probably caused by a lower number of bovid species assembled at chromosome level used in previous publications.

These reconstructed ancestral karyotypes allowed us to identify CRs and EBRs between each ancestor leading to cattle. Our results indicate that chromosome evolution in ruminants is characterized by a first burst of chromosome rearrangements, with a high proportion of interchromosomal ones between RAK and PAK, followed by a period of intrachromosomal reshuffling leading to a final stasis during the past 44 million years (Figures 1b and 2), in line with previous publications (Farré et al., 2019; Kulemzina et al., 2014). For a CR to happen, a DSB must be incorrectly repaired using an unexpected DNA template (reviewed in (Burssed et al., 2022)). The higher rate of CRs between RAK‐PAK (3.69 CRs/My), compared to the most recent branches, might be explained by changes in the DNA repair machinery. As shown in parrots, the loss of genes involved in the repair of DSBs led to an increase of CRs in this species (Huang et al., 2022). Studies on the evolution of the DNA damage repair machinery in ruminants would help to disentangle this issue.

Even if changes in the DSB repair machinery might be linked to the increase in the CR rate, these might not be solely responsible for the type of CR. Instead, the 3D structure of the chromatin within the nucleus might play a role in the potential type of CRs because the template used for repair must be in close physical proximity of the DSB and the repair machinery (Álvarez‐González, Arias‐Sardá, et al., 2022). We recently showed that species whose chromosomes are individually compacted within the nucleus (forming the so‐called chromosome territories) tend to present more interchromosomal rearrangements, while species whose centromeres are all clustered together present more intrachromosomal rearrangements (Álvarez‐González, Arias‐Sardá, et al., 2022). Interestingly, the two types of 3D structures are found within the ruminant clade, with most species showing chromosome territories except for Indian muntjac (Hoencamp et al., 2021), making it temping to speculate that a change in the chromatin 3D structure within the nucleus might have occurred during ruminant evolution.

We observed that CRs and EBRs are not uniformly distributed in cattle chromosomes, with four chromosomes being maintained completely collinear for more than 50 million years (Figure 2). However, the number of EBRs might be underestimated, since EBRs occurring at the ends of cattle chromosomes cannot be detected in our analysis when using cattle as a reference.

EBRs are not associated to regions with high GC content (Figure 3b,c). High GC content is related with recombination hotspots in meiosis due to GC‐biased gene conversion, in which double stranded breaks are preferentially repaired with GC alleles (Arbeithuber et al., 2015). As such, our results point to EBRs happening in open chromatin but not in the same areas as meiotic crossovers. This trend agrees with our most recent findings in rodent species; where EBRs do not co‐localize with meiotic DSBs but with post‐meiotic DSBs in open chromatin regions during male spermatogenesis (Álvarez‐González, Burden, et al., 2022; Burden et al., 2023).

By using transcriptomic data from gene expression atlas in two ruminants and two outgroups, we defined mammalian ancestral housekeeping genes as those orthologous genes labelled as housekeeping in all species, ruminant ancestral housekeeping genes when only present in the two ruminant species while those not shared with any other species were considered cattle‐specific housekeeping genes (Figure 3a). We found that most housekeeping genes are not located in EBRs (Figure 3b,c, Table S5); although not statistically significant. The absence of association could represent a real depletion of housekeeping genes within EBRs or expected by chance given EBRs account for a small proportion of the genome. However, data on carnivores and other mammals have shown that EBRs tend to occur in the boundaries of topologically associated domains suggesting that SFs could be considered regulatory blocks (Álvarez‐González, Burden, et al., 2022; Corbo et al., 2022; Damas et al., 2022). Our findings, combined with previous publications showing changes in expression in genes nearby EBRs (Farré et al., 2019), suggest that only CRs and EBRs not disrupting functional and essential genes became fixed in evolution.

To conclude, we reconstructed the ancestral karyotypes of five ruminants, traced CRs and identified EBRs leading to cattle. Using this dataset, we found that EBRs are not associated with genes, nor housekeeping genes; EBRs are enriched in ruminant‐specific transposable elements but not occurring in high GC‐rich regions. Overall, the emerging picture from our analysis further supports the Integrative Breakage model (Farré et al., 2015) and starts to uncover the evolutionary forces leading to ruminant evolution. Our results open new avenues to investigate CRs in ruminants and their potential role in speciation.

## AUTHOR CONTRIBUTIONS

M.F conceived and devised the study. C.A.‐S., S. Q. and M.F. designed and performed experiments and analysis. C.A.‐S. and M.F wrote the initial first draft of the manuscript with input from S. Q. All authors read and approved the final version of the manuscript.

## FUNDING INFORMATION

We thank Harris Lewin for early access to some of the genome assemblies used herein, and Denis Larkin for early discussions on the data. C.A.‐S. and S. Q. are funded by the GTA fellowship programme from the University of Kent. We thank the specialist High Performance Computing system provided by Information Services at the University of Kent.

## CONFLICT OF INTEREST STATEMENT

The authors declare no conflicts of interest.

## Supporting information


Table S1

Table S2

Table S3

Table S4

Table S5

Figures S1–S5

Data S1

## Data Availability

Data generated in this manuscript are included as supplementary files. Scripts used to analyse the data can be found at https://github.com/Farre‐lab/AriasSarda_ruminants.
