# Supplementary material for: Patterns of chromosome evolution in ruminants
Source: Mol Ecol. 2023 Nov 8;33(24):e17197. doi: 10.1111/mec.17197 (PMC11628655; doi:10.1111/mec.17197)

Supplementary Figure 1

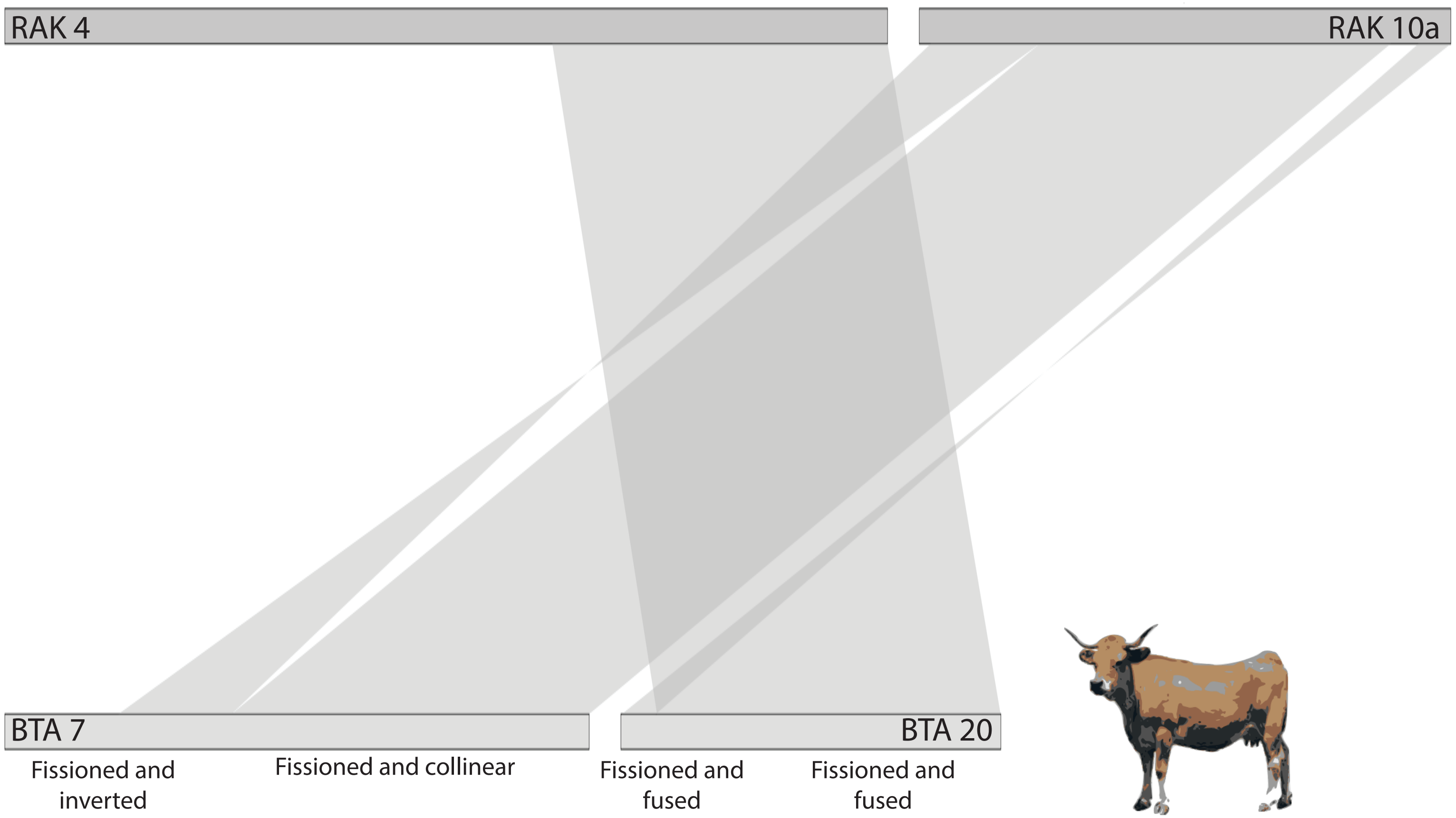

Supplementary Figure 1

Supplementary Figure 2

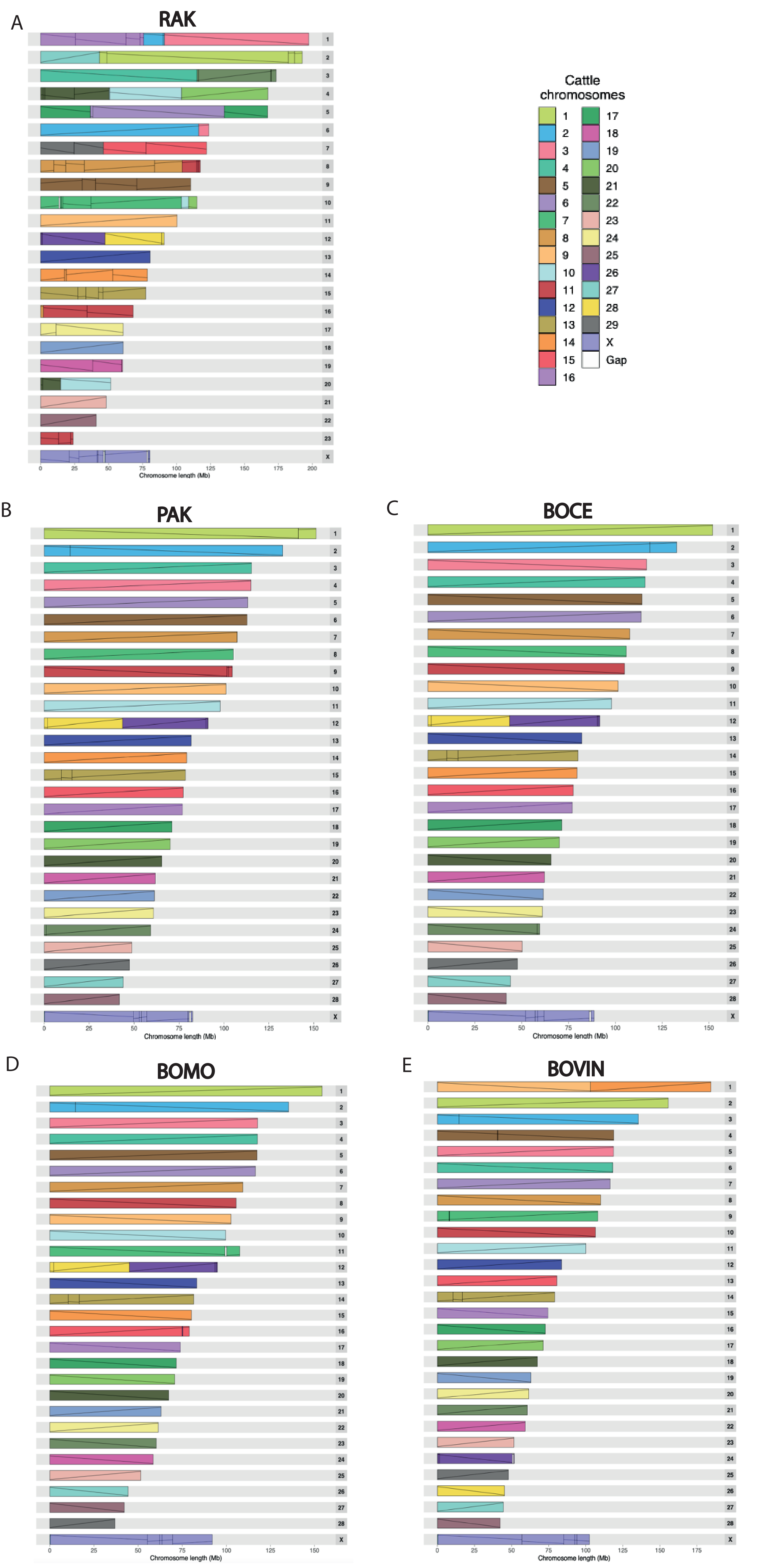

Supplementary Figure 3

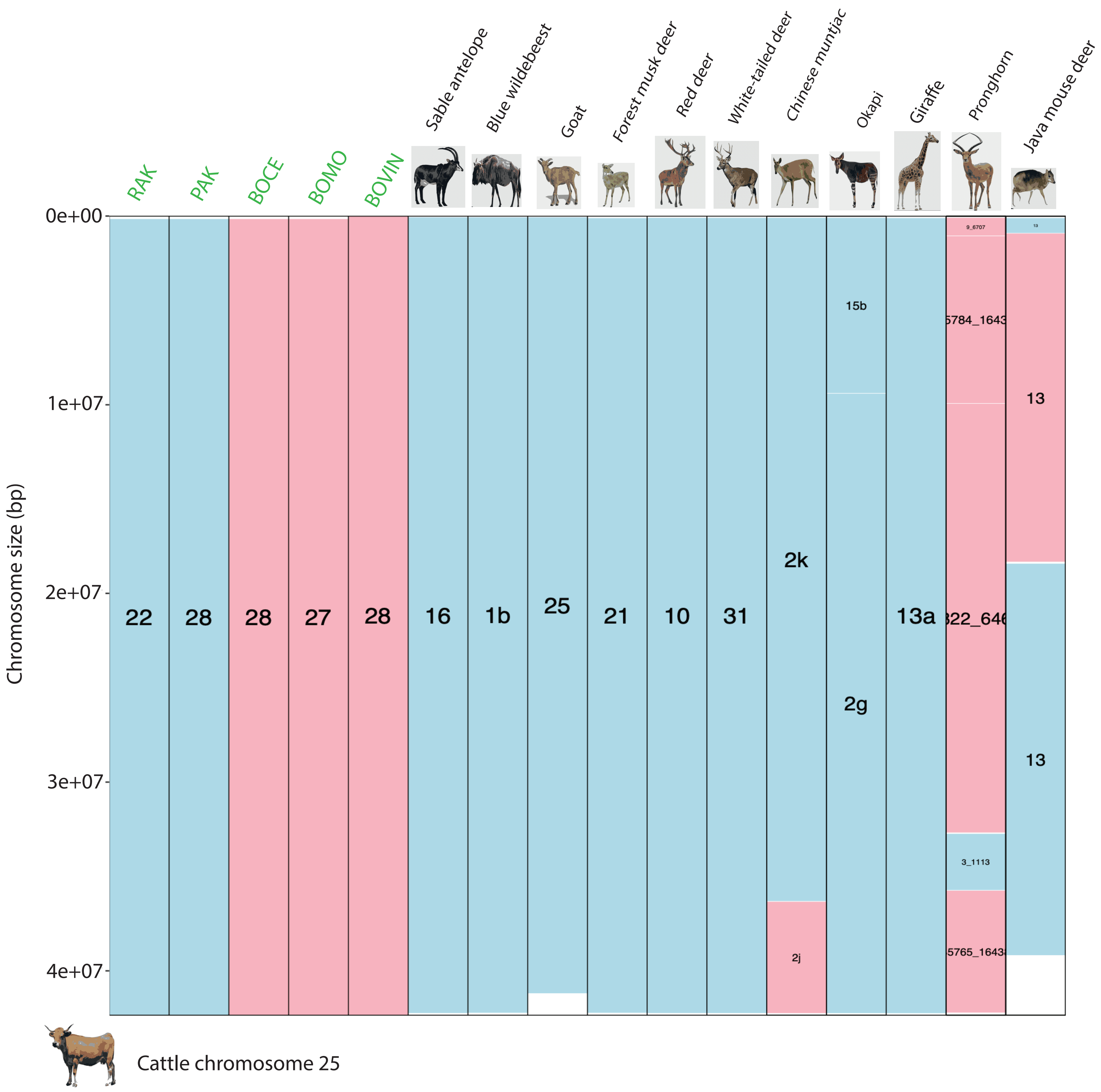

Supplementary Figure 4

A

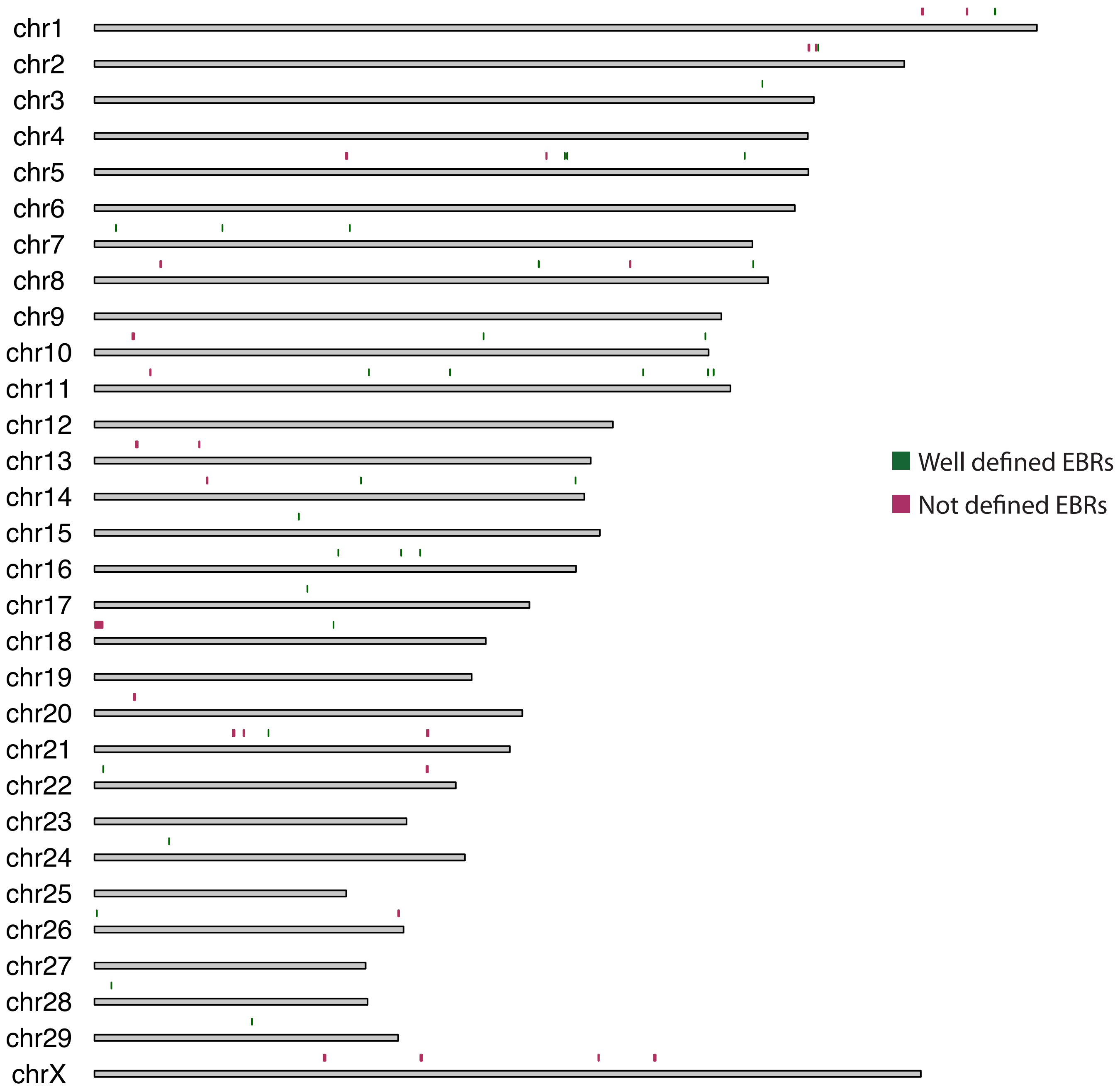

B

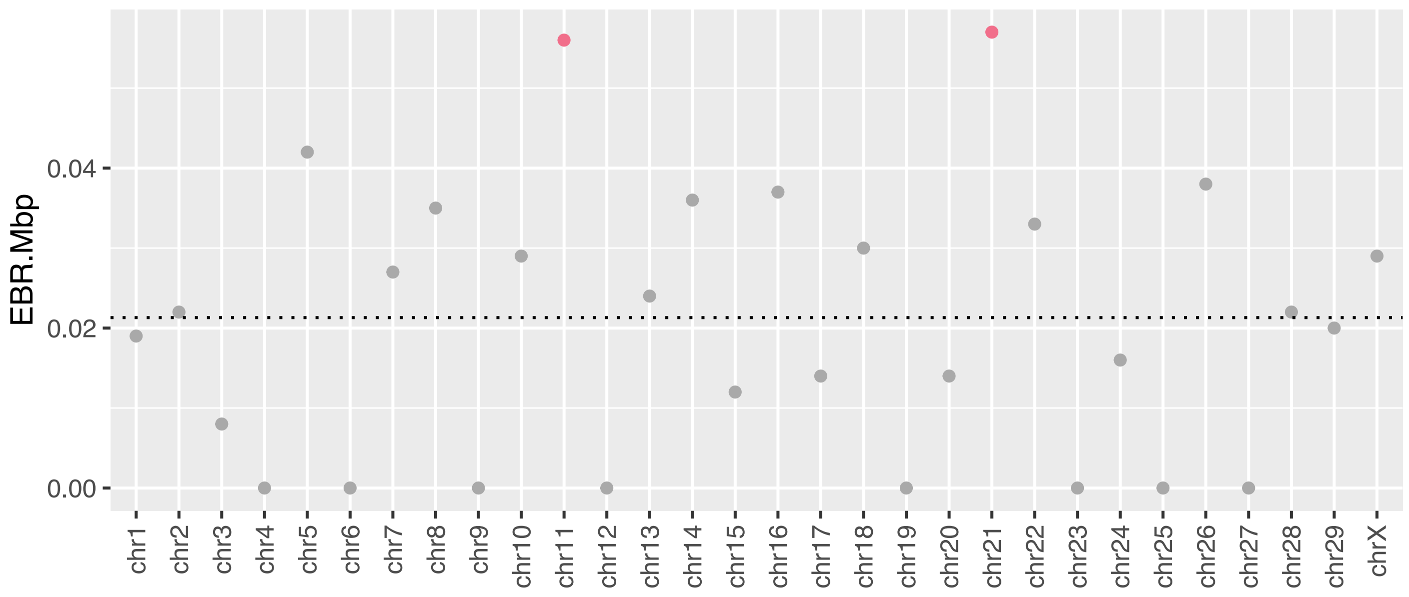

Supplementary Figure 5

A

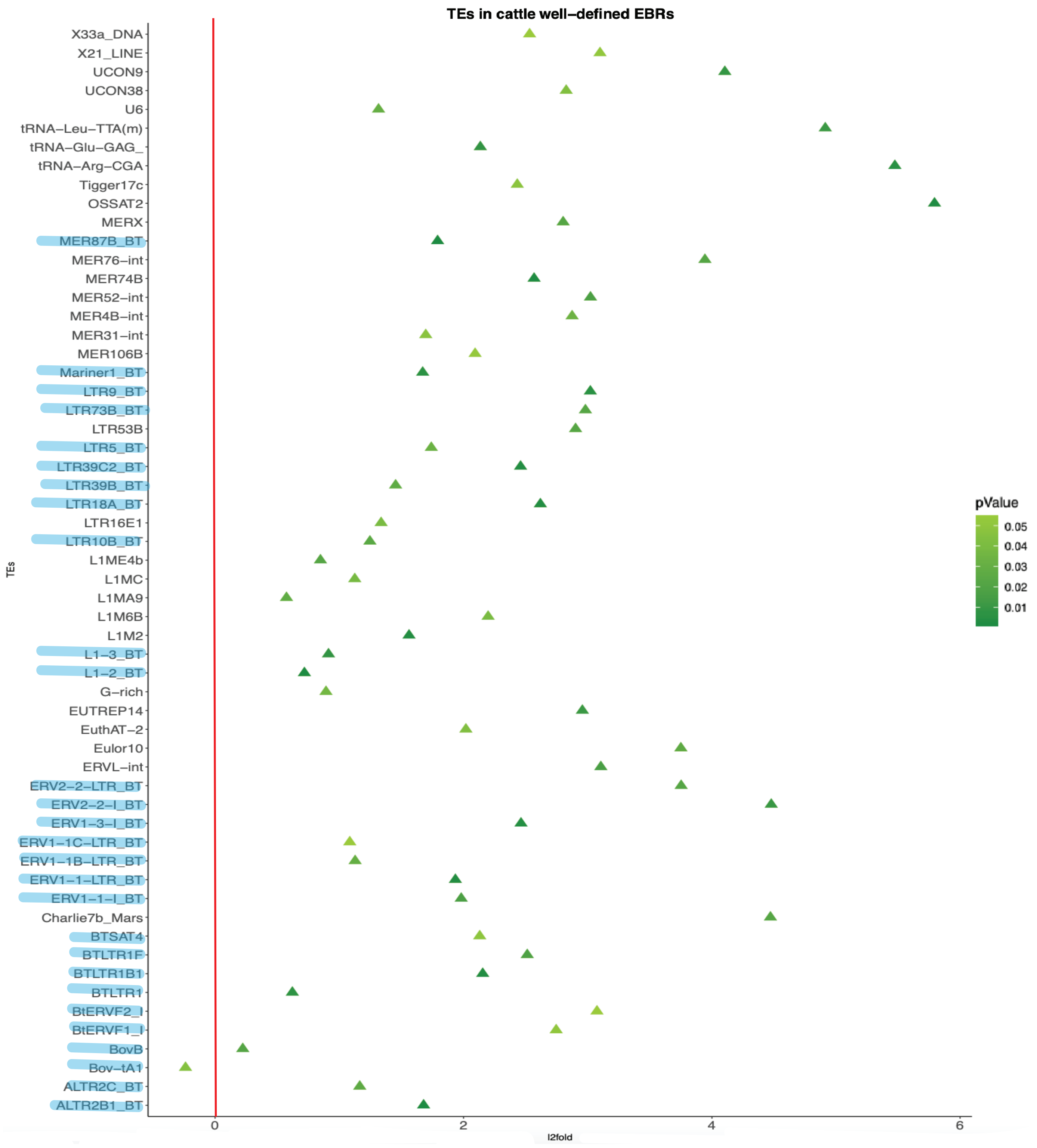

B

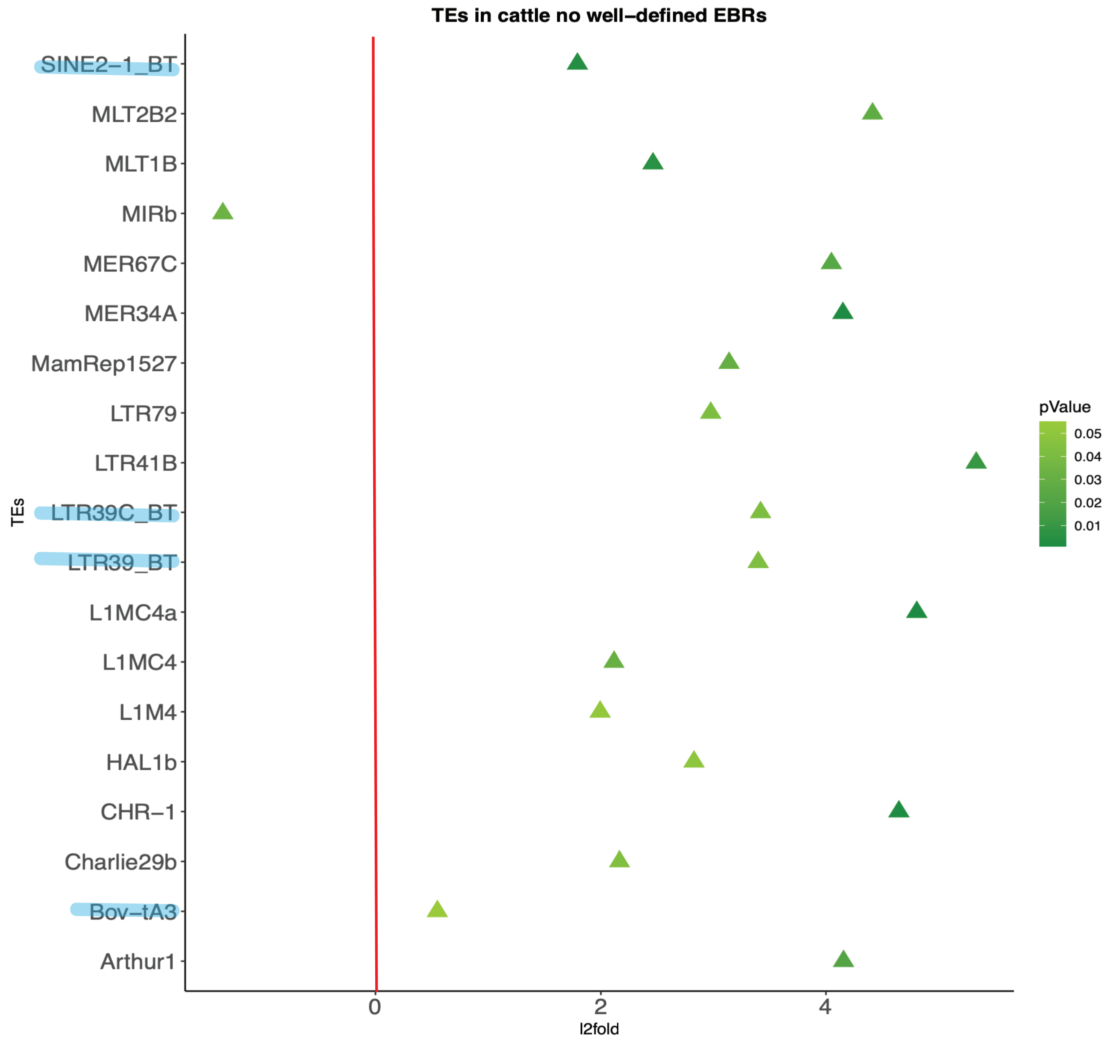

Supplement: Supplementary file 6 — Figures S1–S5 [file MEC-33-e17197-s005.pdf]
