# Supplementary material for: Patterns of chromosome evolution in ruminants
Source: Mol Ecol. 2023 Nov 8;33(24):e17197. doi: 10.1111/mec.17197 (PMC11628655; doi:10.1111/mec.17197)
Supplement: Supplementary file 7 — Data S1 [file MEC-33-e17197-s002.docx]

**SUPPLEMENTARY DATA**

**Supplementary Table 1. List of ruminant species.**

**Supplementary Table 2. Homologies of ancestral karyotypes.** Data used to plot

759 Figure 2.

**Supplementary Table 3. List of cattle EBRs.** Including position, phylogenetic classification and type.

**Supplementary Table 4. List of housekeeping genes in cattle.**

**Supplementary Table 5. Number of genes, transposable elements and GC-rich**

**regions overlapping EBRs or SFs.**
